# Trends in Buprenorphine Prescribing for Opioid Use Disorder by Psychiatrists in the US From 2003 to 2021

**DOI:** 10.1001/jamahealthforum.2023.0221

**Published:** 2023-04-07

**Authors:** Timothy B. Creedon, Mir M. Ali, Zev Schuman-Olivier

**Affiliations:** 1 Office of the Assistant Secretary for Planning and Evaluation, US Department of Health and Human Services, Washington, DC; 2 Department of Psychiatry, Harvard Medical School, Boston, Massachusetts; 3 Center for Mindfulness and Compassion, Cambridge Health Alliance, Cambridge, Massachusetts

## Abstract

This cross-sectional study uses data from a database to compare national trends in patients treated with buprenorphine by psychiatrists and nonpsychiatrists from 2003 to 2021.

## Introduction

Two decades of efforts to increase access to opioid use disorder (OUD) treatment have resulted in substantial increases in the number and kind of clinicians able to prescribe buprenorphine for OUD.^1,2^ Given the high rate of psychiatric comorbidities among patients with OUD (60%),^3^ psychiatrists are uniquely positioned to provide high-quality integrated care for this population.^4^ In the 2010s, however, there were large shifts in buprenorphine prescribing to primary care physicians, physician assistants (PAs), and advanced practice registered nurses (APRNs),^2^ and it is not known how buprenorphine prescribing by psychiatrists has changed since then. This study therefore compared national trends in patients treated with buprenorphine by psychiatrists and nonpsychiatrists from 2003 to 2021.

## Methods

This study followed the relevant portions of the STROBE reporting guideline. Per the Common Rule (45 CFR §46), institutional review board approval was not sought because the study did not involve human participant research. We analyzed cross-sectional data from the IQVIA Total Patient Tracker (TPT) database, January 2003 to December 2021. The TPT database captures the total monthly number of patients across all drugs and clinician specialties dispensed nationally in retail outpatient settings. For each month, TPT reconciles multiple prescription fills per patient, yielding total counts of unique patients within each clinician category. We extracted data on the number of patients with buprenorphine prescription fills for OUD (excluding pain management formulations). To investigate whether changes in buprenorphine prescribing were specific to OUD treatment or associated with broader changes in the field of psychiatry, we also extracted and analyzed the same data for antidepressant fills (eMethods [eTables 1-2] in Supplement 1 lists drugs). We examined trends in the number of patients with fills from psychiatrists vs all other nonpsychiatrist clinicians (eMethods [eTable 3] in Supplement 1 lists clinician types). All analyses were conducted in Stata/MP 17 (StataCorp).

## Results

**Figure 1.**
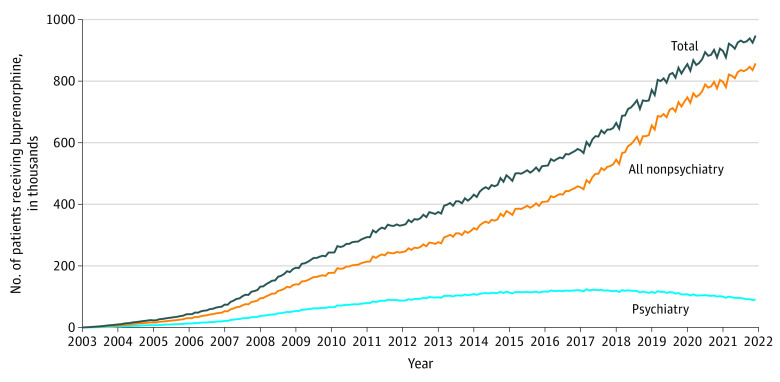
National Trends in the Number of Unique Patients With Buprenorphine Fills, by Prescribing Clinician Specialty From 2003 to 2021 Data are monthly counts (in thousands) of unique patients receiving buprenorphine dispensed in retail outpatient settings, stratified by the type of prescribing clinician. Psychiatry includes psychiatrists and geriatric psychiatrists. Nonpsychiatry includes all other specialties. Total is the sum of both groups.

**Figure 2.**
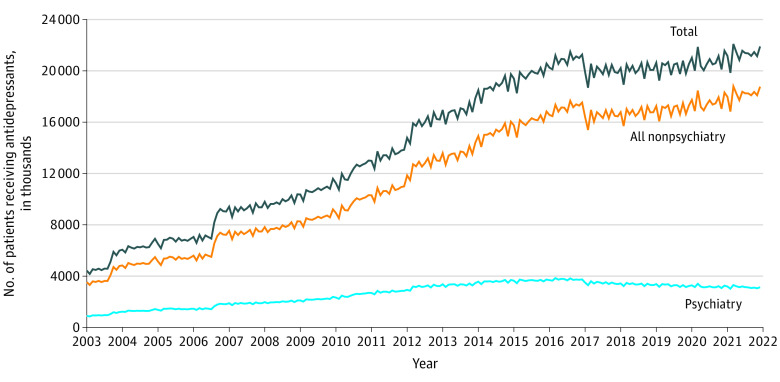
National Trends in the Number of Unique Patients With Antidepressant Fills, by Prescribing Clinician Specialty From 2003 to 2021 Data are monthly counts (in thousands) of unique patients receiving antidepressants dispensed in retail outpatient settings, stratified by the type of prescribing clinician. Psychiatry includes psychiatrists and geriatric psychiatrists. Nonpsychiatry includes all other specialties. Total is the sum of both groups.

From 2003 to 2017, the number of patients with buprenorphine fills from psychiatrists and nonpsychiatrists increased steadily (Figure 1). The proportion of patients with buprenorphine fills from psychiatrists reached a peak in 2004 at 32.2% and then began to decrease. While the number of patients with buprenorphine fills from nonpsychiatrist clinicians continued to increase after 2017, the number of patients with buprenorphine fills from psychiatrists began to decrease in mid-2017. Approximately 124 000 patients received buprenorphine fills from psychiatrists at the high point in March, 2017, but by December, 2021, the number had decreased by 27.4% to approximately 90 000. By the end of 2021, about 10% of patients with buprenorphine fills received them from psychiatrists, representing a decrease of nearly 70% from the proportional high point in 2004 to 2005. Figure 2 shows a similar pattern beginning in 2017 for antidepressant fills prescribed by psychiatrists, suggesting the trend was not unique to buprenorphine.

## Discussion

Psychiatrists have played an important part in providing buprenorphine for OUD since it became available 2 decades ago.^1^ Relative to other specialties, however, psychiatry’s role has reduced over time, especially as primary care physicians, PAs, and APRNs took on more prescribing responsibility in the 2010s.^1,2^ Here we found that, between 2017 and 2021 there was an absolute continuous decrease in the number of patients receiving buprenorphine prescribed by psychiatrists. We also observed that this trend was closely mirrored by the trend for antidepressants.

These data suggest the decrease in buprenorphine prescribing by psychiatrists may not be explained by reasons specific to buprenorphine and OUD treatment but, rather, by growth in both mental health and OUD care by primary care clinicians. Other workforce changes may have also been factors, including a diminishing population of psychiatrists overall,^5^ decreasing acceptance of health insurance among psychiatrists who continue to practice,^6^ and PAs and APRNs being allowed to prescribe buprenorphine since 2016. A limitation of this study is that the data source captured most but not all outlets through which prescription drugs could be dispensed in the US. Small proportions of all buprenorphine and antidepressant prescriptions may have been dispensed elsewhere and therefore not included in this analysis.

Psychiatry can continue to play an important role in the provision of OUD treatment, especially by pairing buprenorphine with other psychiatric services when needed,^4^ but the ability of people with OUD to access treatment for comorbid mental disorders should be closely monitored given the observed shifts in the way care is provided.

## Notes

Supplement 1. **eMethods. eTable 1.** Buprenorphine Formulations for Opioid Use Disorder **eTable 2.** Antidepressants (Generic Names) **eTable 3.** IQVIA Total Patient Tracker (TPT) Provider Specialty Groups

Supplement 2.
Data Sharing Statement
