# Supplementary material for: Trends in Buprenorphine Prescribing for Opioid Use Disorder by Psychiatrists in the US From 2003 to 2021
Source: JAMA Health Forum. 2023 Apr 7;4(4):e230221. doi: 10.1001/jamahealthforum.2023.0221 (PMC10082400; doi:10.1001/jamahealthforum.2023.0221)
Supplement: Supplement 1. — eMethods. eTable 1. Buprenorphine Formulations for Opioid Use Disorder eTable 2. Antidepressants (Generic Names) eTable 3. IQVIA Total Patient Tracker (TPT) Provider Specialty Groups [file jamahealthforum-e230221-s001.pdf]

Supplemental Online Content

Creedon TB, Ali MM, Schuman-Olivier Z. Trends in buprenorphine prescribing for opioid use disorder by psychiatrists in the US from 2003 to 2021. *JAMA Health Forum.* 2023;4(4):e230221. doi:10.1001/jamahealthforum.2023.0221

eMethods. eTable 1. Buprenorphine Formulations for Opioid Use Disorder

eTable 2. Antidepressants (Generic Names)

eTable 3. IQVIA Total Patient Tracker (TPT) Provider Specialty Groups

This supplemental material has been provided by the authors to give readers additional information about their work.

© 2023 Creedon TB et al. *JAMA Health Forum*.

eMethods

eTable 1. Buprenorphine Formulations for Opioid Use Disorder

BUNAVAIL 11/2014 BID
BUPRENORPHIN/NALOX AI&
BUPRENORPHIN/NALOX QYC
BUPRENORPHIN/NALOX 01/2018 SPI
BUPRENORPHIN/NALOX 02/2016 AKO
BUPRENORPHIN/NALOX 02/2017 A8K
BUPRENORPHIN/NALOX 03/2013 AM9
BUPRENORPHIN/NALOX 03/2013 TEV
BUPRENORPHIN/NALOX 03/2018 MKR
BUPRENORPHIN/NALOX 06/2013 AS&
BUPRENORPHIN/NALOX 06/2016 G.M
BUPRENORPHIN/NALOX 06/2018 DRL
BUPRENORPHIN/NALOX 07/2014 HKM
BUPRENORPHIN/NALOX 08/2013 AVK
BUPRENORPHIN/NALOX 09/2018 LNN
BUPRENORPHINE HCL AI&
BUPRENORPHINE HCL DI/
BUPRENORPHINE HCL PXM
BUPRENORPHINE HCL 01/2015 AS&
BUPRENORPHINE HCL 02/2018 R/P
BUPRENORPHINE HCL 04/2015 MYN
BUPRENORPHINE HCL 05/2010 TEV
BUPRENORPHINE HCL 05/2017 SPI
BUPRENORPHINE HCL 07/2011 AKO
BUPRENORPHINE HCL 10/2009 HKM
BUPRENORPHINE HCL 10/2010 M3Y
BUPRENORPHINE HCL 10/2012 QYC
PROBUPHINE 09/2016 BBH
SUBLOCADE 03/2018 IDV
SUBOXONE 01/2003 IDV
SUBUTEX 03/2003 R&B
ZUBSOLV 09/2013 OEX
BUPRENORPHIN/NALOX 02/2019 ALE
BUPRENORPHIN/NALOX 02/2019 MYN
BUPRENORPHIN/NALOX 02/2019 SDZ
BUPRENORPHIN/NALOX A0P
BUPRENORPHINE AM9
BUPRENORPHINE QYC
BUPRENORPHINE 06/2017 TEV
BUPRENORPHINE 07/2017 R/P
BUPRENORPHINE HCL A-A
BUPRENORPHINE HCL FG6
BUPRENORPHINE HCL HWK
BUPRENORPHINE HCL PNO
BUPRENORPHINE HCL PYI
BUPRENORPHINE HCL 03/2010 LCM
BUPRENORPHINE HCL 05/2015 BBP
BUPRENORPHINE HCL 06/1997 PFZ
BUPRENORPHINE HCL 06/2005 BDF
BUPRENORPHINE HCL 07/2013 MD6
BUPRENORPHINE HCL 08/2015 P.H

© 2023 Creedon TB et al. *JAMA Health Forum*.

eTable 2. Antidepressants (generic names)

Amitriptyline
Amoxapine
Bupropion
Citalopram
Clomipramine
Desipramine
Desvenlafaxine
Doxepin
Duloxetine
Escitalopram
Fluoxetine
Fluvoxamine
Imipramine
Isocarboxazid
Levomilnacipran
Maprotiline
Milnacipran
Mirtazapine
Nefazodone
Nortriptyline
Paroxetine
Phenelzine
Rasagiline
Selegiline
Sertraline
Tranylcypromine
Trazodone
Trimipramine
Venlafaxine
Vilazodone
Vortioxetine

© 2023 Creedon TB et al. *JAMA Health Forum*.

eTable 3. IQVIA Total Patient Tracker (TPT) Provider Specialty Groups

**Psychiatrist**	
Specialty Group Description	*TPT Label*
Geriatric Psychiatry	PYG
Psychiatry	PSY

**Non-Psychiatrist**	
Specialty Group Description	*TPT Label*
Addiction Medicine	ADM
Allergy	ALLR
Anesthesiology	ANES
Cardiology	CARD
Cardiothoracic Surgery	CTS
Cardiovascular Surgery	CVS
Clinical & Lab Imm, Aller & Imm	ALI
Clinical & Lab Immunology, IM	ILI
Clinical & Lab Immunology, PED	PLI
Dermatological Immunology	DDL
Clinical Pharmacology	CN
Critical Care Medicine, IM	CCM
Critical Care, Neuro Surgery	NCC
Critical Care, Ob/Gyn	OCC
Colon & Rectal Surgery	CRS
Dentistry	DENT
Dermatology	DERM
Dermatopathology	DMP
Emergency Medicine	EM
Emergency Medicine	EM
Endocrinology	ENDO
Family Practice	FP
Gastroenterology	GE
General Practice	GP
General Practice	GPM
General Surgery	GS
Genetics	GEN
Geriatrics	GER
Hematology	HEM
Hepatology	HEP
Hospice & Palliative Medicine	HPM
Infectious Diseases	ID
Internal Medicine	IM
Internal Medicine/Pediatrics	MPD
Medical Microbiology	MM
Naturopaths	ND
Nephrology	NEPH

© 2023 Creedon TB et al. *JAMA Health Forum*.

**Non-Psychiatrist**	
Specialty Group Description	*TPT Label*
Neurological Surgery	NS
Neurology	NEUR
Nuclear Medicine	NM
Nurse Practitioner	NRP
Nutrition	NTR
Ob/Gyn	OBG
Occupational Medicine	OM
Oncology	ONC
Ophthalmology	OPH
Optometry	OPT
Orthopedic Surgery	ORS
Orthopedic Surgery of the Spine	OSS
Osteopathic Medicine	DO
Other	OS
Other Surgery	SURG
Otolaryngology	OTO
Otology	OT
Pain Medicine	PMD
Pathology	PTH
Pediatric Critical Care Medicine	CCP
Pediatric Neurological Surgery	NSP
Pediatrics	PED
Physical Med & Rehabilitation	PM
Physician Assistants	PHA
Pharmacists	PHR
Plastic Surgery	PLS
Podiatry	POD
Psychology	PYA
Pulmonary Critical Care Med	PCC
Pulmonary Diseases	PUD
Radiology	RAD
Rheumatology	RHU
Sleep Medicine	SM
Sports Medicine	SPM
Surgical Critical Care Medicine	CCS
Thoracic Surgery	THS
Urology	UROL
Unspecified Specialty	UNSP
Veterinary Medicine	VET

© 2023 Creedon TB et al. *JAMA Health Forum*.
