# Supplementary material for: Trends in Buprenorphine Prescribing for Opioid Use Disorder by Psychiatrists in the US From 2003 to 2021
Source: JAMA Health Forum. 2023 Apr 7;4(4):e230221. doi: 10.1001/jamahealthforum.2023.0221 (PMC10082400; doi:10.1001/jamahealthforum.2023.0221)
Supplement: Supplement 2. — Data Sharing Statement [file jamahealthforum-e230221-s002.pdf]

Data Sharing Statement

Creedon. Trends in Buprenorphine Prescribing for Opioid Use Disorder by Psychiatrists in the US From 2003 to 2021. *JAMA Health Forum*. Published April 07, 2023. doi:10.1001/jamahealthforum.2023.0221

Data
Data available: No

Additional Information
Explanation for why data not available: The study data (IQVIA Total Patient Tracker) are proprietary. We do not have permission to make the data available.
